# Long-term survival following SCLC transformation after osimertinib resistance in *EGFR*-mutant NSCLC: Benefit from local surgical resection and osimertinib rechallenge

**DOI:** 10.1016/j.gendis.2026.102129

**Published:** 2026-03-06

**Authors:** Qian Zhang

**Affiliations:** a Department of Respiratory Medicine, Pinghu First People's Hospital, Jiaxing, Zhejiang 314200, China; b Department of Thoracic Medical Oncology, Zhejiang Cancer Hospital, Hangzhou, Zhejiang 310022, China

Transformation to small cell lung cancer (T-SCLC) is a resistance mechanism in *EGFR*-mutant non-small cell lung cancer (NSCLC), primarily occurring in patients with lung adenocarcinoma harboring sensitive *EGFR* mutations who develop resistance after epidermal growth factor receptor (EGFR)-tyrosine kinase inhibitor (TKI) therapy. The incidence ranges from 5% to 14%, typically emerging 14–26 months (median 18 months) after EGFR-TKI treatments.^1^ Most T-SCLCs retain the original lung adenocarcinoma driver mutations (∼84%–88%) while acquiring classic SCLC genetic features, such as TP53 and RB1 mutations. Existing therapeutic approaches for T-SCLC are primarily supported by evidence from retrospective studies and case reports. Chemotherapy remains the most common treatment option for T-SCLC. Although most T-SCLC cases initially respond well to etoposide–platinum combination chemotherapy, the duration of response is typically limited.^1^ This case report demonstrates a viable therapeutic alternative for this patient subset, supporting the role of surgery and targeted therapy rechallenge in T-SCLC transformation.

A 54-year-old female nonsmoker was referred to our hospital in April 2016 with a 2-month history of cough. On April 20, 2016, she underwent radical surgery for right lung adenocarcinoma (stage: pT3N0M0, IIB). Adjuvant chemotherapy consisting of gemcitabine (1000 mg/m^2^, intravenously, every 3 weeks) plus cisplatin (80 mg/m^2^, intravenously, every 3 weeks) was administered from May to July 2016 (4 cycles total). In December 2017, a follow-up positron emission tomography-computed tomography (PET-CT) revealed pleura and right third anterior rib metastases (rT0N0M1b, IVA). Genetic test of the postoperative tissue sample confirmed *EGFR* exon 19 deletion, prompting gefitinib (250 mg, orally, daily) on December 15, 2017, achieving a partial response (PR). Slow progression (rib lesion enlargement) led to local radiotherapy (May–June 2019). On June 1, 2019, plasma genetic testing detected *EGFR* T790M, prompting a switch to osimertinib (80 mg, orally, daily) with sustained PR.

On September 19, 2022, the patient reported worsening right-sided chest pain. Contrast-enhanced chest computed tomography (CT) demonstrated progression of the rib metastatic lesion. Given that the lesion had progressed after prior radiotherapy and presented as an isolated, resectable metastasis, a multidisciplinary team discussion recommended surgical resection to achieve optimal local cytoreduction and to obtain a definitive histopathological specimen, rather than pursuing repeat radiotherapy or ablation. Subsequently, the patient underwent surgical resection of the chest wall rib lesion on September 30, 2022. Histopathological examination of the surgical specimen confirmed transformation to T-SCLC. The patient chose to continue osimertinib (80 mg, orally, daily) after localized surgery with regular follow-ups. As of the latest follow-up, the patient has achieved a progression-free survival (PFS) of over 29 months, with a performance status of 0. A summary of the patient's diagnosis and treatment history is shown in Figure 1.

**Figure 1:**
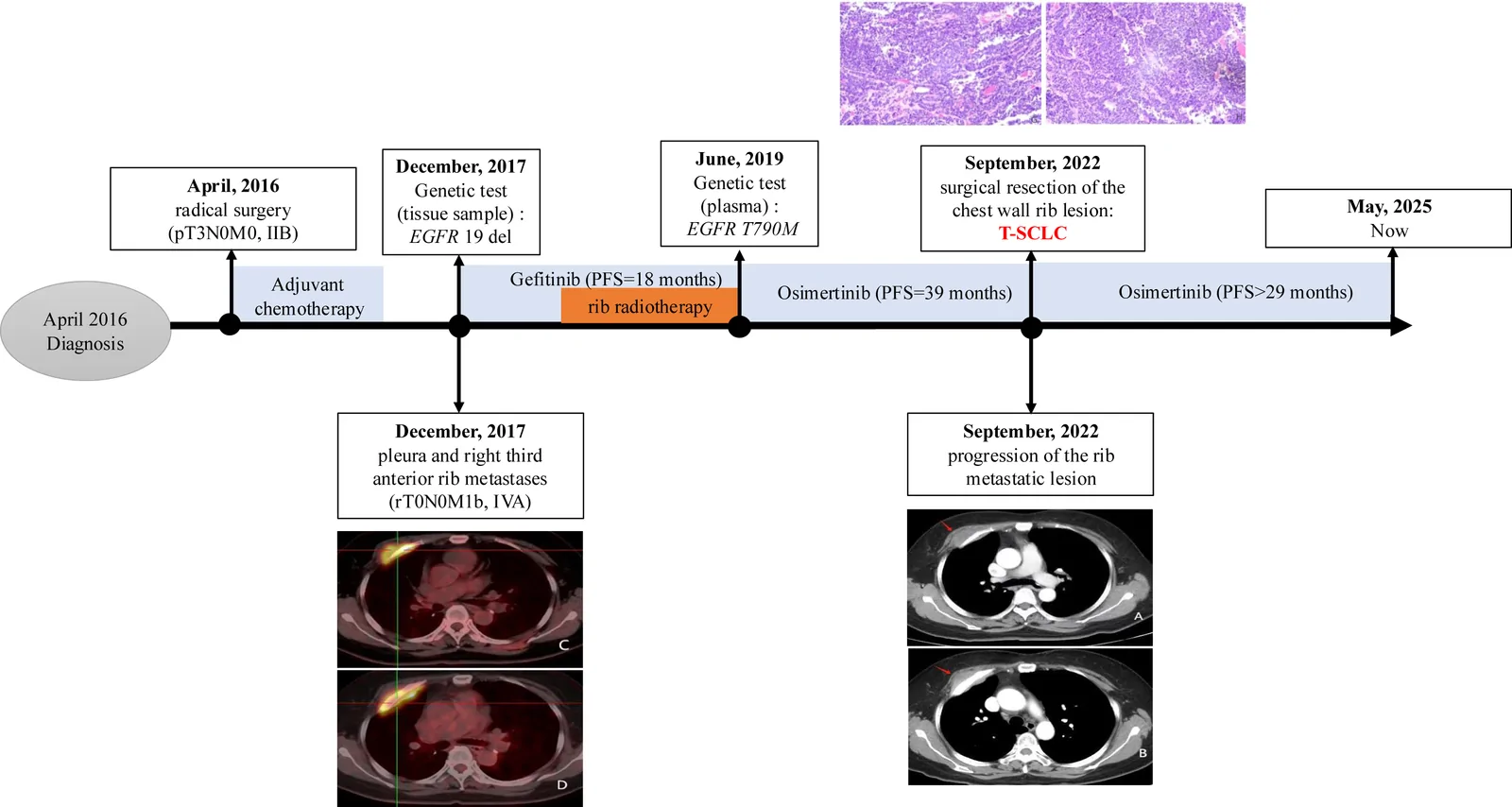
The timeline of the patient's treatment and diagnostic history.

This case offers novel clinical insights into the management of T-SCLC following EGFR-TKI resistance, demonstrating the potential efficacy of combined surgical intervention and targeted therapy rechallenge, which is markedly surpassing the median overall survival (OS) (10–20 months) typically observed in T-SCLC patients.^2^ Currently, there is a lack of prospective randomized controlled studies to guide treatment strategies for T-SCLC. In previous retrospective studies, the median PFS was only 3.4 months (95% CI, 2.4–5.4 months) among 46 T-SCLC patients treated with etoposide–platinum chemotherapy. Moreover, research on combining other drugs with chemotherapy is still under investigation. However, the efficacy remains controversial, and the data rely exclusively on retrospective studies. This case demonstrates a characteristic oligometastatic progression pattern, with all recurrences limited to indolent right chest wall rib metastases following surgery and targeted therapy. Palliative metastasectomy provided effective cytoreduction and extended disease control (PFS: 29 months), highlighting the therapeutic value of local intervention in selected patients with slow-progressing, anatomically confined recurrences. Notably, serial monitoring of neuroendocrine markers revealed persistently normal levels of neuron-specific enolase (NSE; peak 19.20 ng/mL) and gastrin-releasing peptide (GRP; peak 67.40 pg/mL) throughout the disease course, including during the histologically confirmed T-SCLC phase. The successful rechallenge with osimertinib and the achievement of long-term disease control in this case may be attributed to tumor clonal heterogeneity, the elimination of resistant clones through local intervention, and the persistent dependency on the EGFR pathway post-transformation. First, T-SCLC typically retains the original EGFR mutation but is often accompanied by inactivation of RB1 and TP53, which are key drivers of neuroendocrine phenotypic transformation. However, several studies suggest that the transformation process may be incomplete or regionally heterogeneous, with some tumor cells maintaining EGFR dependency.^3^^,^^4^ For example, the research by Offin et al has confirmed that in EGFR-mutant lung cancers, co-deletion of RB1 and TP53 is significantly associated with the risk of transformation, yet sensitive EGFR mutations remain detectable in the patient post-transformation, and their response to subsequent TKI therapy is closely related to clonal architecture.^3^ Second, this case achieved complete resection of the metastatic lesion through surgery. In oligometastatic lung cancer, local radical therapies have been proven to alter disease progression and extend survival by reducing tumor burden, limiting the dissemination of resistant clones, and creating a more favorable microenvironment for systemic therapy.^5^ Third, the patient's serum neuroendocrine markers (NSE and GRP) remained consistently normal throughout the disease course, suggesting that the transformation may represent low-grade neuroendocrine differentiation or incomplete phenotypic transition, distinct from the biological behavior of classical SCLC. The sustained absence of neuroendocrine marker elevation may provide a partial biological rationale for the observed clinical benefit from osimertinib rechallenge, suggesting retained EGFR-dependence despite histological transformation.

Several limitations of this report warrant consideration. First, the findings are based on a single case with a unique presentation of an isolated, slow-growing rib metastasis, which limits generalizability. Second, the lack of TP53/RB1 status analysis at transformation hinders a complete understanding of the co-mutational landscape. Future prospective studies incorporating extensive molecular characterization are needed to validate this approach and identify predictive biomarkers.

## CRediT authorship contribution statement

**Qian Zhang:** Writing – review & editing, Writing – original draft, Validation, Conceptualization.

## Ethics declaration

This retrospective case report analysis was conducted after the patient's treatment. The study protocol was approved by the Ethics Committee of Zhejiang Cancer Hospital (IRB-2024-1325), ensuring compliance with ethical standards for research involving human subjects. Written informed consents were obtained from the patient and his family members.

## **Conflict of** interests

The author declare that they have no known competing financial interests or personal relationships that could have appeared to influence the work reported in this paper.
