# Supplementary material for: Long-term survival following SCLC transformation after osimertinib resistance in EGFR-mutant NSCLC: Benefit from local surgical resection and osimertinib rechallenge
Source: Genes Dis. 2026 Mar 6;14(1):102129. doi: 10.1016/j.gendis.2026.102129 (PMC13542495; doi:10.1016/j.gendis.2026.102129)
Supplement: Supplementary file 1 [file mmc1.doc]

**Supplementary Materials and Methods**

1. Clinical History and Diagnosis

1.1 Patient Presentation

A 54-year-old female non-smoker presented in April 2016 with a 2-month history of cough. She had no significant past medical history or family history of malignancy.

1.2 Initial Diagnosis and Staging (April 2016)

Surgical Procedure: The patient underwent a radical right lower lobectomy with systematic lymph node dissection (stations 2, 4, 7, 10, 11, 12) on April 20, 2016.

Pathological Assessment: The resected specimen was diagnosed as a poorly differentiated lung adenocarcinoma, predominantly solid pattern, measuring 5x5x4 cm, with invasion into the bronchial cartilage. Pathological stage was pT3N0M0 (Stage IIB) according to the AJCC 8th edition. All sampled lymph nodes were negative for metastasis (0/8). Immunohistochemistry (IHC) staining of the primary tumor was performed using standard protocols. Results were as follows: AE1/AE3 (+), p63 (focal+), TTF-1 (+), CK5/6 (-), NapsinA (+), CK7 (+), Syn (-), CgA (-), Ki-67 (approximately 80%), p40 (-), ALK (D5F3) (-), EGFR (+). This profile confirmed the diagnosis of lung adenocarcinoma without neuroendocrine differentiation.

1.3 First Recurrence and Systemic Therapy (December 2017)

Imaging Diagnosis: Disease recurrence was suspected based on new right anterior chest wall pain. A contrast-enhanced chest CT (December 15, 2017) and PET-CT (December 13, 2017) confirmed an isolated osteolytic lesion with a soft-tissue mass in the right 3rd anterior rib (SUVmax=14.6), consistent with oligometastatic disease (rT0N0M1b, Stage IVA).

Molecular Testing: To identify the targetable driver mutation, the primary tumor tissue was subjected to next-generation sequencing (NGS). The results revealed a sensitizing EGFR exon 19 deletion.

Systemic Treatment: Based on the molecular findings, first-line therapy with gefitinib (250 mg orally, once daily) was initiated on December 15, 2017. Zoledronic acid (4 mg intravenously every 4 weeks) was administered concurrently for bone metastasis.

2. Response Evaluation and Follow-up Protocols

Tumor response was assessed according to the Response Evaluation Criteria in Solid Tumors (RECIST) version 1.1. Contrast-enhanced chest and abdominal CT scans were performed every 1 months. Brain MRI with contrast was performed every 6 months, or when clinically indicated by neurological symptoms. Complete blood count, liver and renal function tests were performed monthly during chemotherapy and every 1 months during TKI therapy. Serum tumor markers, specifically Neuron-Specific Enolase (NSE) and Gastrin-Releasing Peptide (GRP), were measured every 1 months using electrochemiluminescence immunoassays (Roche Diagnostics). Reference ranges were <16.3 ng/mL for NSE and <80 pg/mL for GRP.

3. Management of Disease Progression

3.1 First Progression (May-June 2019)

Pattern of Progression: Slow, isolated enlargement of the known right rib metastasis was observed on serial CT scans, meeting criteria for oligoprogression.

Molecular Testing: The EGFR T790M mutation was identified via plasma-based NGS testing. This assay did not evaluate other resistance mechanisms or tumor suppressor genes.

Local Therapy: The symptomatic rib lesion was treated with palliative external beam radiotherapy. Systemic Therapy Change: Following confirmation of T790M, systemic therapy was switched to osimertinib (80 mg orally, once daily) in June 2019.

3.2 Second Progression and Histologic Transformation (September 2022)

Pattern of Progression: The patient presented with worsening right-sided chest pain. Imaging confirmed progression of the previously irradiated rib lesion and a new small nodule in the left lower lobe.

Multidisciplinary Team (MDT) Decision: Given the oligoprogressive pattern, a MDT comprising thoracic surgery, radiation oncology, medical oncology, and radiology recommended surgical resection. The goals were: 1) to achieve maximal local cytoreduction for a symptomatic, isolated progressing lesion; 2) to obtain ample tissue for definitive histopathological and molecular analysis.

Surgical Procedure: On September 30, 2022, the patient underwent thoracoscopic partial resection of the left lower lobe for diagnostic removal of the suspicious metastatic nodule, concurrently combined with an extended resection of the chest wall rib tumor (specifically, en bloc resection of the right 3rd anterior rib metastasis involving the adjacent chest wall soft tissue and pleura). The primary surgical objective was to achieve macroscopic complete (R0) resection of the chest wall metastasis while obtaining a pathological diagnosis of the pulmonary nodule.

Pathological Diagnosis of Transformation:

Histology & IHC: The resected rib tumor showed morphological features of a poorly differentiated neuroendocrine carcinoma (small cell type), admixed with a minor component of adenocarcinoma. IHC was performed using standard automated platforms (e.g., Ventana BenchMark). The staining profile was: Synaptophysin (Sy) (+), CD56 (+), TTF-1 (+), Ki-67 (approximately 80%), NapsinA (-), p40 (-), p63 (-), CK7 (+), CAM5.2 (+). This confirmed transformation to small cell lung cancer (T-SCLC). The left lung nodule was benign (organizing inflammation).

4. Post-Transformation Treatment and Follow-up

4.1 Postoperative Systemic Therapy

Based on evidence-based medical recommendations developed through MDT, and taking into account the patient's personal preferences regarding quality of life, treatment convenience, and tolerability, the patient continued maintenance osimertinib.

4.2 Outcome Assessment

Primary Outcome: Progression-free survival (PFS) was defined as the time from treatment initiation to the first disease progression, death, or the last follow-up..

Status: At the last follow-up in May 2025, the patient remained progression-free for over 29 months, with an Eastern Cooperative Oncology Group (ECOG) performance status of 0.
